# Verbal memory in major depressive disorder in a long-term perspective: a five-year longitudinal study of first episode patients

**DOI:** 10.3389/fpsyt.2025.1623126

**Published:** 2025-07-03

**Authors:** Marit Schmid, Eivind Haga Ronold, Maiken Løchen, Åsa Hammar

**Affiliations:** ^1^ Faculty of Health and Social Sciences, Department of Welfare and Participation, Western Norway University of Applied Sciences, Bergen, Norway; ^2^ Division of Psychiatry, Haukeland University Hospital, University of Bergen, Bergen, Norway; ^3^ Department of Biological and Medical Psychology, University of Bergen, Bergen, Norway; ^4^ Mohn Medical Imaging and Visualization Centre, Department of Radiology, Haukeland University Hospital, Bergen, Norway; ^5^ Department of Psychiatry, Molde Hospital, Møre og Romsdal Hospital Trust, Molde, Norway; ^6^ Department of Clinical Sciences Lund, Psychiatry, Faculty of Medicine, Lund University, Lund, Sweden; ^7^ Department of Psychiatry, Skåne University Hospital, Lund, Sweden

**Keywords:** major depression, first episode depression, cognitive deficits, longitudinal, relapse

## Abstract

**Introduction:**

Major depressive disorder (MDD) is associated with cognitive impairment, including verbal memory. Limited knowledge exists following memory performance in first episode (FE) MDD. This study investigated verbal memory, depressive symptoms, and relapse in FE MDD over five years, from the trait, state, and scar perspectives. These perspectives suggests that deficits in memory either preexists, manifest in MDD, or exacerbates with every MDD episode, respectively.

**Methods:**

Thirty patients and 30 healthy matched controls (HC) were assessed using the California Verbal Learning Test, second edition (CVLT-II) across three test occasions; in the acute phase (Y0), at one-year (Y1) and five-year (Y5) follow-up. The relationship between CVLT-II scores and depression severity (measured by the Montgomery Åsberg Depression Rating Scale) and relapse at the five-year follow-up, was assessed.

**Results:**

The FE MDD group demonstrated significantly poorer performance on List A, Trial 1 immediate free recall at Y0 compared to HC, however correction for multiple comparisons the difference did not reach significance. No differences were observed in any other condition at any time point. Further, the PG had a significant improvement on List A, trial 1 from Y0 to Y5. No associations were found between symptom severity and verbal memory, and no performance differences were identified between patients with and without relapse in a five -year perspective.

**Discussion:**

In conclusion, individuals with FE MDD show normal verbal memory performance, but exhibit impaired performance on List A, Trial 1 immediate free recall in the acute phase improving in remission, indicative of a state-related deficit in auditory attention. No evidence of scarring deficits in the FE MDD group was observed in the follow-up period.

## Introduction

Major depressive disorder (MDD) is a common mental disorder. Estimated lifetime prevalence is between 15,3%-16,6% (1, 2), and at any time, 5% of the population suffers from depression (3). MDD is associated with lower quality of life (4), psychosocial impairment (5), impaired daily functioning (6) and occupational impairment (7, 8). Notably, improvement in depressive symptoms does not automatically yield improvement in daily functioning, and impairment may persist in remission, even ten years post onset (9), making the individual vulnerable to new episodes of MDD.

MDD is associated with a high recurrence. Over 50% of patients experience new episodes within two years of onset (10), and between 50% and 85% of patients will experience multiple episodes throughout their life course (11). Residual symptoms and previous episodes increase the risk of relapse and recurrence (12). The consequences of MDD are substantial on individual and societal levels, particularly due to its relapsing remitting nature. Therefore, enhancing the understanding of how MDD develops from first onset is crucial.

Cognitive impairments are frequently reported in the acute phase of MDD, recognized as a central component of the disease in several reviews (13, 14) and meta-analysis (15), with potential impacts on quality of life (16) and daily functioning (17). Subjective reports of cognitive impairment often exceed results found in neurocognitive tests (18, 19). However, studies of neurocognitive tests have demonstrated that over 50% of patients with MDD show general cognitive impairment in the acute phase (20, 21), which persists after recovery from mood symptoms (22, 23), and could contribute to relapse risk (24, 25). In contrast, other reviews report improvement and even normalization of cognitive functioning in remission (14, 26), or intact cognitive performance irrespective of illness state (27, 28).

The diverse findings may be attributed to variations in age (29, 30), education (31, 32), premorbid intelligence (32, 33), categorization of the depressed group (34), depression severity (35), duration of illness and number of episodes (36), effects of medication (37, 38) psychological treatment (39), debut (23, 40), and comorbid disorders (34). Differences in test characteristics and definitions of impairment may also contribute (27, 33). According to memory performance motivation has been proposed as an additional factor influencing performance in MDD (41). Given the heterogeneity of depressive symptoms, some even propose discrete neurocognitive subgroups in MDD (42–44).

According to subgroups of depression, deficits manifest across various domains in both first episode (FE) (45–48) and recurrent and remitted MDD (49–51). However, there is limited knowledge regarding the existence of such impairment prior to the onset of MDD and their potential impact on subsequent illness (52). Thus, investigating FE MDD may provide insights into how deficits develop.

Verbal memory impairment have previously been found both in the acute phase (14, 53, 54) and in remission (51, 55), although some studies report no verbal memory impairment (56, 57). Impairment in immediate and delayed free recall have been found (58), while recognition and cued recall to a greater extent seems to be intact (56, 58). Hammar and Årdal (59) found intact verbal memory performance with *repeated material* presentation, indicating slower learning, but intact recall and recognition with repetition. A recent large-scale analysis investigating verbal memory in several neurological and psychiatric groups, showed no memory impairment in MDD (60). However, this study did not differentiate between subgroups of depression or follow patients in a longitudinal perspective.

Limited knowledge exists about the longitudinal development of verbal memory function in MDD, and results vary. Differences in FE and recurrent MDD have been noted (45, 54, 56, 61), suggesting performance exacerbation with each episode (14, 23). Intact verbal memory have been found in FE MDD (56, 61). However studies have also shown impaired verbal memory in this group (45, 47, 48), with improvements in remission (26, 62). Thus, investigating an ecologically relevant domain of memory, such as verbal memory, in a clearly defined subgroup with FE MDD in a longitudinal follow-up could advance the understanding of the neurocognitive profile in MDD.

The neurocognitive profile in MDD could be understood through three perspectives (22). In the first perspective, the neurocognitive impairment could be pre-existing (trait) markers, and be present prior to diagnosis, representing an underlying vulnerability (63, 64), or be persistent during periods of symptomatic remission, independent of clinical state (64–67). In the second perspective impairment the impairments are found to be caused by mood (state) symptoms, and temporary linked to the current mood state, but normalizing in symptom reduction and remission (68, 69), noting differences in cognitive function between fully remitted, partially remitted, and acute-phase MDD patients (70). In the third perspective deficits are understood as caused by neurobiological changes associated with the stress following the depressive symptoms. The *scar* perspective suggests such enduring and progressively worsening deficits associated with the number and duration of episodes (71–76), with an association reported between HPA-axis dysregulation and impaired memory function in recurrent MDD (77), but not in FE MDD (78). MDD is often associated with acute or chronic stress (79), with evidence of elevated cortisol levels and impaired cortisol suppression in depressed individuals (73, 80). These abnormalities are associated with impaired memory and hippocampal atrophy (81–84). The current literature is however non-conclusive regarding the validity of any specific perspective regarding verbal memory. Thus, it is valuable to investigate verbal memory performance in FE MDD in a longitudinal perspective.

### The present study

To the authors knowledge, this is the first study investigating verbal memory longitudinally in a five-year follow-up in FE MDD. The main aim of the present study was to investigate verbal memory in a group of FE MDD patients acutely, and how verbal memory function develops longitudinally compared to a well-matched healthy control group.

The following research questions were investigated:

1. Will the patient group (PG) show impaired verbal memory functioning compared to the healthy control group (HC) in the acute phase of illness, Y0?
2. If present, will poor performance in verbal memory functioning in the acute phase of the illness in the PG persist or improve in a longitudinal perspective?
3. Is there an association between verbal memory functioning and depression severity?
4. Is there a relationship between verbal memory functioning and relapse experience across time?

Based on previous research we expect that the PG will show impaired verbal memory functioning compared to the HC in the acute phase of the illness. Further, we hypothesize that cognitive impairment in verbal memory will improve in a five year perspective, and that the patient group will not differ from the HC in both follow-up assessments. Further, we expect there to be a positive association between symptom severity and cognitive impairment in verbal memory. Furthermore, we want to explore if patients who had a relapse within the five years performed worse in verbal memory function in the acute phase of illness.

## Materials and methods

### Design

This was a longitudinal case control study with follow-up from Y0 at baseline in the acute phase of illness, Y1 at one-year follow-up, and Y5 at five-year follow-up.

### Recruitment and participant flow

**Figure 1:**
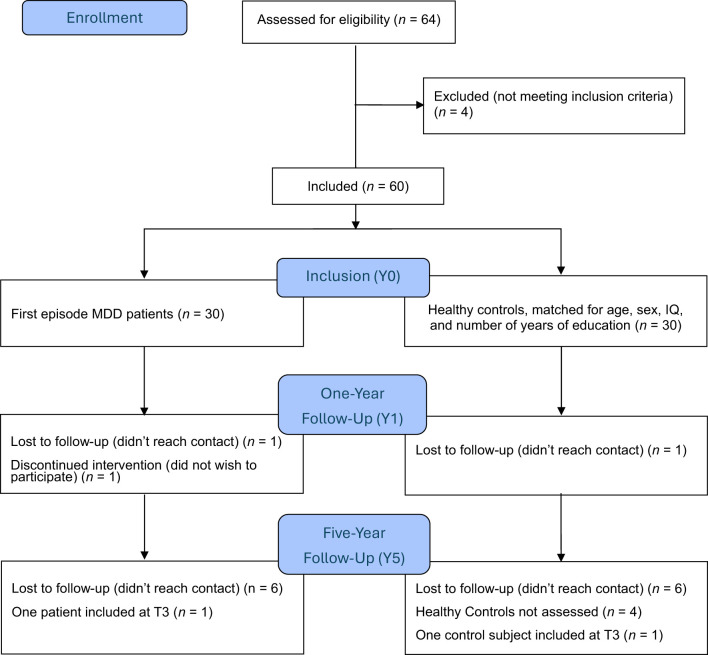
Participant flowchart.

A total of 60 participants were included in the study (Y0), consisting of 30 patients; 16 males and 14 females, and 30 healthy individually matched controls (see flowchart in **Figure 1** and **Table 1** for demographic data). Demographics and procedures have been described in previous reports by Ronold et al. (85) and Schmid & Hammar (25).

**Table 1:** Clinical and demographic variables for the Patient Group (PG) and the Healthy Control Group (HC).

Variables Y0	PG (*n* = 30)		HC (*n* = 30)		Statistics	
	*M*	*SD*	*M*	*SD*	*t*	*p*
Age	26.20	5.94	26.17	5.69	0.02	.98 ^a^
Male/Female	16/14	NA	16/14	NA	NA	NA
Education (years)	13.97	1.71	14.03	1.65	-0.15	.88 ^a^
Total IQ (WASI)	118.53	8.12	120.97	8.23	−1.15	.25 ^a^
MADRS	24.60	3.79	NA	NA	NA	NA

Variables Y1	PG (*n* =28)		HC (*n* = 29)			
Age	26.93	5.33	26.83	5.11	0.73	.94
Male/Female	14/14	NA	14/15	NA	NA	NA
Education (years)	14.29	1.76	14.79	1.66	-1.12	.28
Total IQ (WASI)	118.43	8.4	121.31	8.15	-1.31	.19
MADRS	10.10	5.95	NA	NA	NA	NA
N Dep	8	28,57%	NA	NA	NA	NA

Variables Y5	PG (*n* =23)		HC (*n* = 20)		Statistics	
Age	30.34	5.74	30.45	6.09	-.057	.96
Male/Female	11/12	NA	9/11	NA	NA	NA
Education (years)	15.35	2.35	16.60	2.01	-1.86	.07
Total IQ (WASI)	118.65	8.47	119.7	8.27	-0.41	.69
MADRS	8.87	8.14	NA	NA	NA	NA
N Dep	7	30.43%	NA	NA	NA	NA

PG, patient group; HC, healthy control group; NA, not applicable; WASI, Wechsler Abbreviated Scale of Intelligence; MADRS, Mongomery and Åsberg Depression Rating Scale.

^a^ Independent samples t-test. N Dep = number and percentage of participants with MADRS ≥ 12.

Patients were enrolled in the study through cooperation with doctors and psychologists in primary healthcare. Inclusion criteria for the PG were a diagnosis of single episode MDD according to the Diagnostic and Statistical Manual of Mental Disorders- Forth Edition, Text Revision (86), using the Mini-International Neuropsychiatric Interview (MINI), (87), and a minimum score of 20 on the depression symptom questionnaire, Montgomery Åsberg Depression Rating Scale (MADRS) (88), indicating moderate to severe depression. Exclusion criteria was previous diagnosis of depression or having received treatment for depression previously, psychosis, neurological conditions and somatic disorders that could influence cognition, alcohol or substance abuse, or treatment with electroconvulsive therapy.

The HC was recruited through acquaintances of employees at the department matched to the patient group with respect to age, gender, and years of education (within a +/- 2-year limit). Three patients were excluded due to a history of recurrent MDD, and one patient was excluded because of Norwegian language difficulties.

### Clinical characteristics

Two patients received a co-morbid diagnosis of panic disorder with agoraphobia. Fourteen patients were prescribed antidepressant medication: twelve with selective serotonin reuptake inhibitors, one with serotonin noradrenaline reuptake inhibitor, and one with tetracyclic antidepressant. All patients were outpatients. Four patients received only medical treatment, nine received only psychological treatments, ten received both medical and psychological treatments. At Y5 the National Institute of Mental Health Life Chart Method (89) was used to retrospectively assess relapse of MDD. Relapse was defined as reporting one or more depressive episodes since Y0, and % with depression ≥ 12 on MADRS (see **Table 1**).

### Ethics

The study was performed in accordance with the Helsinki Declaration of the World Medical Association Assembly. The Regional Committee for Medical Research Ethics and the Norwegian Data Inspectorate approved the study (REK 23218). Informed consent was obtained from all participants.

### Neuropsychological assessment

The California Verbal Learning Test, second edition (CVLT-II) (90), was used to measure verbal memory. The test entails presentation of a list of words and measures the following cognitive functions: acquisition with immediate free recall List A, learning (list 1-5), short- and long-term memory, and recognition. In total, seven of the variables from the CVLT-II were analyzed in the present study: (a) immediate free recall, List A, trial 1; (b) learning, trial 1-5, a sum score for correct responses in all five trials; (c) short delay free recall; (d) short delay cued recall; (e) long delay free recall; (f) long delay cued recall; and (g) total hits delayed recognition. High score on either of these variables indicated good performance.

All participants completed the Wechsler Abbreviated Scale of Intelligence (WASI), (91) with the Two-Subtest Form consisting of vocabulary and matrix reasoning. The assessments were conducted at an outpatient neuropsychology clinic at the university.

### Analyses and statistics

Preliminary assumption testing was conducted to check for any statistical violations and the data were examined for outliers. For all analyses, a specified significance level of *p* ≤.05 was used, with 95% CI. Eta-squared (η^2^) was employed as the measure of effect size, and Bonferroni method was used to correct for multiple comparisons in analyses. Independent samples t-tests were computed to compare the PG and the HC with regard to age, years of education, and IQ scores. ANOVA was performed to compare PG and HC on the different CVLT-II conditions (raw scores) at Y0, Y1 and Y5, and the independent variable was group. To control for multiple comparisons in this analysis Bonferroni correction of α-values was implemented (.05/21 = .002). For the immediate free recall, trial 1 condition, a mixed between-within subjects’ analysis of variance with three time points was performed to compare the groups according to change in performance across Y0, Y1, and Y5. To explore potential exacerbation a *change score* from Y0 to Y5 was calculated for the immediate free recall trial 1 condition for all patients and controls, and an independent samples t-test was performed to compare the PG and the HC according to change score. To further explore performance across time, a paired-samples t-test was computed to analyze the change in performance separately for the PG and the HC from Y0 to Y5. To control for multiple comparisons on both these analyses, Bonferroni correction of α-values was implemented (.05/14 = .004). The relationship between CVLT-II scores and severity of depression symptoms (measured by MADRS) was examined for the PG by Pearson’s correlation at all time points, Y0, Y1, and Y5 (α-values for each correlation matrix was corrected for multiple comparison (.05/6 = .006). The PG were also divided according to the experience of relapse during the years since initial episode which resulted in a relapse group (*n* = 16) and a no relapse group (*n* = 6). Independent samples t-tests were computed to compare the relapse group with the no relapse group in performance on the immediate free recall trial 1 condition of the CVLT-II at Y0, Y1, and Y5 and according to MADRS score at Y0, Y1, and Y5, and on the change score in performance from Y0 to Y5.

## Results

### Matching of groups

Independent samples t-tests showed that the PG and the HC did not differ with regard to age, years of education, and total IQ at Y0, Y1 or Y5 (see **Table 1**). Inspection of mean MADRS score showed that the PG was in remission at Y1 and Y5 (see **Table 1**).

### CVLT-II performance

#### Verbal memory in the acute phase of illness, T1

**Figure 2:**
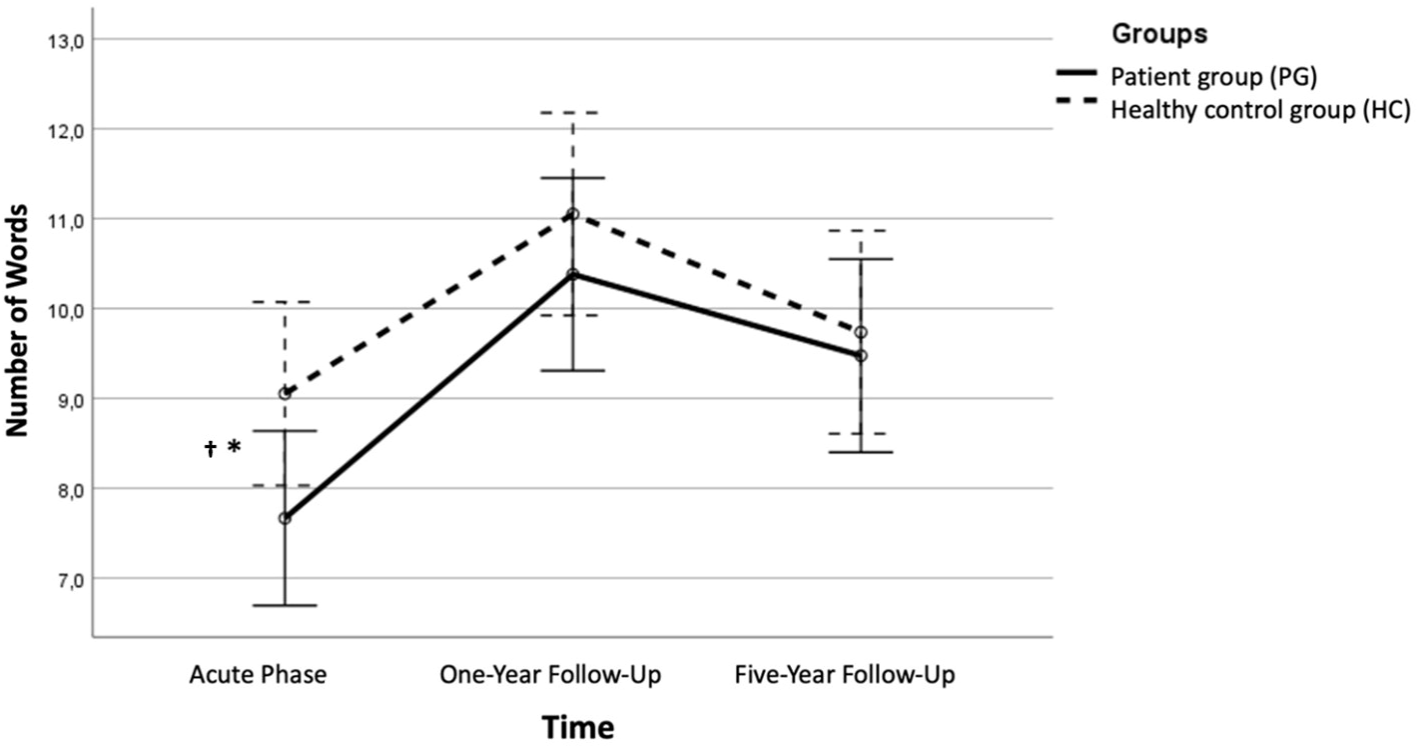
The immediate free recall condition 1 across time. Error bars represent 95% CIs. Significant difference between the PG and the HC in performance on the first trial, immediate free recall *p < .05 † Bonferroni corrected alpha value (> .002).

Investigating the differences between the PG and the HC on the conditions of the CVLT-II, the ANOVA showed that the PG performed significantly poorer compared to the HC on the first trial on CVLT-II, immediate free recall, trial 1at Y0. However, this result was not significant when adjusting for multiple comparisons (> *p*.002). The two groups did not differ in the other CVLT-II conditions at Y0 or at any other time point (see **Table 2** for raw scores and statistics; and **Figure 2** for an illustration).

**Table 2:** CVLT-II scores for the PG and the HC at Y0, Y1, and Y5.

Time	Y0					Y1					Y5				
	PG (*n* = 30)	HC (*n* = 30)	Statistics			PG (*n* = 28)	HC (*n* = 29)	Statistics			PG (*n* = 23)	HC (*n* = 20)	Statistics		
Outcome	*M* (SD)	*M* (SD)	*F* (df)	*p*	η^2^	*M* (SD)	*M* (SD)	*F* (df)	*p*	η^2^	*M* (SD)	*M* (SD)	*F* (df)	*p*	η^2^
CVLT-II															
IFR TR 1	**7.53 (2.18)**	**9.17 (2.82)**	**6.31 (1)**	.015* †	**.10**	10.32 (1.98)	10.97 (2.40)	1.22 (1)	.275	.02	9.61 (2.25)	9.80 (2.53)	0.07 (1)	.794	.00
LTR 1-5	60.73 (8.47)	64.93 (8.63)	3.62 (1)	.062	.06	68.79 (6.24)	68.55 (8.33)	0.01 (1)	.905	.00	67.78 (6.20)	68.05 (8.67)	0.01 (1)	.907	.00
SDFR	13.68 (2.29)	14.07 (2.03)	0.51 (1)	.478	.01	14.89 (1.60)	14.86 (1.90)	0.00 (1)	.948	.00	14.91 (1.53)	14.85 (1.50)	0.02 (1)	.893	.00
SDCR	13.97 (2.24)	14.23 (2.05)	0.23 (1)	.632	.00	15.14 (1.11)	15.21 (1.63)	0.03 (1)	.864	.00	15.30 (1.02)	15.30 (1.13)	0.00 (1)	.989	.00
LDFR	14.20 (2.30)	14.63 (2.03)	0.60 (1)	.441	.01	15.00 (0.98)	15.17 (1.49)	0.26 (1)	.609	.01	15.17 (1.03)	15.20 (1.24)	0.01 (1)	.940	.00
LDCR	14.37 (2.07)	14.57 (1.88)	0.15 (1)	.698	.00	15.25 (1.04)	15.27 (1.73)	0.01 (1)	.946	.00	15.44 (0.79)	15.35 (1.09)	0.09 (1)	.769	.00
THDR	15.73 (0.69)	15.73 (0.83)	0.00 (1)	1.000	.00	15.71 (0.60)	15.66 (0.86)	0.09 (1)	.765	.00	15.83 (0.49)	15.85 (0.37)	0.03 (1)	.859	.00

Means and statistics for the condition yielding statistically significant results are shown in bold. Gender is presented as (male/female). PG, patient group; HC, healthy control group; IFR TR 1, immediate free recall trial 1; LTR 1-5, Learning trial 1-5; SDFR, short delay free recall; SDCR, short delay cued recall; LDFR, long delay free recall; LDCR, long delay cued recall; THDR, total hits delayed recognition.

*p <.05. † Bonferroni corrected alpha value (>.002).

#### Verbal memory across time

At Y1 and Y5, when patients were in symptom reduction and remission, the ANOVA showed that the PG and the HC did not differ in their performance in any of the CVLT-II conditions (see **Table 2** for raw scores and statistics and **Figure 2** for an illustration).

##### The immediate free recall, trial 1 condition, across time

The mixed between-within subjects analysis of variance with three time points showed no significant effect between groups, Λ = .93, *F (2*, 37) = 1.20, *p* = .313, η^2^ = .06. There was a significant main effect of time, Λ = .48, *F*(2, 37) = 19.89, *p* <.001, η^2^ = .52, indicating a large effect, showing that across time there was a significant improvement in scores for both groups (see **Figure 2**). An independent samples t-test showed that the change score from Y0 to Y5 for the PG (*M* = 2.0, *SD* = 2.44) did not reach statistical significance compared to HC(*M* = 0.68, *SD* = 2.47), *t*(39) = 1.71, *p* = .096 (two-tailed). The mean score difference was 1.32, 95% CI [-0.24, 2.87], with a medium effect size η^2^ = .07. A paired-samples t-test showed that the PG had a significant improvement from Y0 (*M* = 7.59, *SD* = 1.79) to Y5 (*M* = 9.59, *SD* = 2.30), *t*(21) = -3.83, *p* <.001 (two-tailed). This finding was also significant when adjusting for multiple comparisons (Bonferroni correction *p* <.007). The mean increase in scores on the immediate free recall trial 1 condition was 2.0, 95% CI [-1.29, 3.68], η^2^ = .41, indicating a large effect. For the HC, a paired-samples t-test showed that there was no significant change in scores from Y0 (*M* = 9.05, *SD* = 2.57) to Y5 (*M* = 9.74, *SD* = 2.58), *t*(18) = -1.21, *p* = .243 (two-tailed). The mean increase in scores on the immediate free recall trial 1 condition was 0.68, 95% CI [-0.73, 0.19], η^2^ = .07.

#### Verbal memory and depression severity

At Y0 and Y1, a Pearson’s correlation analysis showed no significant correlations between MADRS score and performance in the different conditions on the CVLT-II. At Y5, there was a significant negative correlation between MADRS score and performance on the total hits delayed recognition condition – the higher MADRS score the poorer performance on the condition, however, this association was not significant following Bonferroni correction for multiple comparisons (p >.006). There were no other significant correlations between MADRS score and performance on the CVLT-II (see **Table 3**).

**Table 3:** Correlations between CVLT-II conditions and MADRS scores for the PG at Y0, Y1, Y5.

Variable T1	*n*	*M*	*SD*	1	2	3	4	5	6	7	8
1. MADRS	30	24.60	3.73	—							
2. IFR TR 1	30	7.53	2.18	.18	—						
3. LTR1-5	30	60.43	8.47	.17	.68**	—					
4. SDFR	30	13.67	2.29	.17	.35	.79**	—				
5. SDCR	30	13.97	2.24	.10	.49**	.87**	.84**	—			
6. LDFR	30	14.20	2.30	.12	.52**	.90**	.83**	.882**	—		
7. LDCR	30	14.37	2.08	.16	.47**	.85**	.85**	.946**	.93**	—	
8. THDR	30	15.73	0.69	.04	.33	.68**	.68**	.641**	.82**	.70**	—
Variable T2	*n*	*M*	*SD*	1	2	3	4	5	6	7	8
1. MADRS	28	9.89	6.05	—							
2. IFR TR 1	28	10.32	1.98	.10	—						
3. LTR 1-5	28	68.77	6.24	.15	.84**	—					
4. SDFR	28	14.89	1.59	.09	.35	.60**	—				
5. SDCR	28	15.14	1.11	.00	.33	.57**	.76**	—			
6. LDFR	28	15.00	.98	-.05	.29	.53**	.71**	.88**	—		
7. LDCR	28	15.25	1.04	-.05	.23	.45*	.71**	.85**	.93**	—	
8. THDR	28	15.71	.60	.10	.27	.42*	.93**	.68**	.82**	.70**	—
Variable T3	*n*	*M*	*SD*	1	2	3	4	5	6	7	8
1. MADRS	23	9.38	8.33	—							
2. IFR TR 1	23	9.61	2.25	-.02	—						
3. LTR 1-5	23	67.78	6.20	-.06	.73**	—					
4. SDFR	23	14.91	1.53	-.03	.54**	.81**	—				
5. SDCR	23	15.30	1.02	.07	.49*	.80**	.95**	—			
6. LDFR	23	15.17	1.03	-.01	.50*	.63**	.82**	.88**	—		
7. LDCR	23	15.44	.79	-.08	.61**	.81**	.79**	.85**	.80**	—	
8. THDR	23	15.83	.49	-.48*†	-.02	.12	.28	.68**	.33	.32	—

Bonferroni correction estimate at significance level <.001. Correlation between CVLT-II conditions and MADRS score yielding statistically significant results are shown in bold. IFR TR 1, immediate free recall trial 1; LTR 1-5, Learning trial 1-5; SDFR, short delay free recall; SDCR, short delay cued recall; LDFR, long delay free recall; LDCR, long delay cued recall; THDR, total hits delayed recognition.

*p <.05. **p <.01 † Bonferroni corrected alpha value (>.006).

#### Verbal memory and relapse

Dividing the PG according to relapse experience from inclusion and across time, from Y0 to Y5, independent samples t-tests revealed no significant differences in performance on the immediate free recall trial 1 condition at Y0, Y1 or Y5. The independent samples t-test investigating the change score from Y0 to Y5 showed that it was no significant difference between the patients that experienced relapse and those that did not (See appendix 1 for statistics and means).

## Discussion

The main aim of this study was to investigate verbal memory in a group of FE MDD patients longitudinally in a five-year perspective compared to a well-matched healthy control group. The results showed that the PG performed poorer compared to the HC in one of the conditions on the CVLT-II in the acute phase, the immediate free recall trial 1 condition. No differences in performance on the CVLT-II were found between the groups on the other conditions, or at any conditions at Y1 or Y5. The results further showed that the PG had a significant improvement in performance across time, and showed a more pronounced improvement compared to the control group. No association between verbal memory functioning and symptom severity was found, except on total hits delayed recognition, where those with higher symptom severity performed worse. Finally, when dividing the PG according to relapse experience at Y5, no differences in cognitive performance or symptom severity were found between the relapse and the no relapse group at any time point.

### Verbal memory across time

In the acute phase, patients with FE MDD performed significantly worse than the HC only in the first trial of the CVLT-II, immediate free recall trial 1. However, this result was not significant when correcting for multiple comparisons and must be interpreted with caution Thus, the performance in the other CVLT-II conditions indicated relatively intact memory functioning. These results do not support the hypothesis of verbal memory deficit in the acute phase of illness, but rather show that the patients perform worse on the first trial, indicating deficits in acquisition and auditory attention, affecting a small component of the verbal learning process in the depressed state.

The findings align with some previous studies pointing to first trial immediate free recall trial 1 as a particularly vulnerably function (58, 59). No memory impairment was found in conditions other than immediate free recall trial 1, indicating a general intact verbal memory function, aligning with studies reporting intact verbal memory function in the acute phase of FE MDD (56, 61). However, although the results did not reveal substantial impairment, which patients themselves commonly report (18, 19), the results can still be seen as reflecting the memory outcomes reported by patients, given that everyday memory function is dependent on auditory attention, in the acquisition phase. Hammar and Årdal (74) reported similar results in recurrent MDD, showing impaired performance only in immediate free recall trial 1, with intact function when the material is presented more than once, possibly indicating an auditory attention deficit rather than a memory impairment aligning with several other studies reporting impaired attention in the acute phase of depression (58, 59). Contradiction our findings however, the large- scale study on verbal memory in MDD did not find impairment in the first trail, trial 1 on the CVLT-II test (60), and found intact performance in verbal memory in total, showing the contradicting results concerning verbal memory performance in MDD. Importantly, one could also understand the impairment in auditory attention to be caused by stress, anxiety and motivational factors since trial 1, lit A represent the first encounter with a challenging task.

However, in contrast to Hammar and Årdal’s (59) findings with prevailed impairment in immediate free recall in recurrent MDD patients, the present study suggests a normalization in auditory attention in remission. This may point to a difference in trajectory of memory in FE MDD and recurrent MDD patients. The present results demonstrated that the PG showed a significant improvement in performance over time, from inclusion to follow-up, and performed at the same level as the HC in both follow-up assessments, indicating normalization in auditory attention in phases of symptom reduction and remission. The findings suggest that verbal memory is not impaired in FE MDD patients, and that their impaired auditory attention function will improve in symptom reduction and remission in a five-year perspective.

MADRS scores indicated that the PG was in remission at Y1 and Y5. In contrast to previous studies indicating persisting verbal memory impairment during symptom reduction (20, 92–94) or partial improvement in remission (40, 95, 96), the present findings align with reviews and meta-analysis reporting normalized memory function in remission in FE MDD (26, 62).

The absence of further verbal memory impairment beyond the first trial measuring auditory attention, along with the normalization of function, may be explained by individual- and clinical factors in the sample and point to a difference in memory function between FE and recurrent MDD. The present sample comprises young outpatients with normal IQ, all being FE MDD patients with limited illness duration. Despite a mean MADRS score indicating moderate to severe depression, the cut-off at 20 can still be considered relatively low. The sample may therefore represent a sub-group with minor cognitive impairment, implying a need only for minor improvement in remission to reach normal functioning. Moreover, age is of particular importance. Higher age is associated with increased vulnerability to depression related cognitive impairment, including memory (29, 31). The vulnerability may be explained by age-related changes in neurobiological mechanisms related to depression, interacting with depression-related changes in the same mechanisms potentially creating a cumulative effect (54). Further, higher age is associated with increased severity and chronicity of the illness (29) which can lead to progressively worsening of cognition (22). Following this, the present sample appears too young and relatively healthy compared to other depressed populations to fully demonstrate these dynamics.

### Verbal memory and depression severity

A significant negative correlation emerged between MADRS score and performance on the total hits delayed recognition condition, indicating a very limited association between depression severity and verbal memory impairment, where higher symptom severity is associated with more impaired memory. This association was not significant after correcting for multiple comparisons. Thus, no systematic correlation was found between depression severity and cognitive performance, and the hypothesis postulating a positive association between symptom severity and cognitive impairment was not confirmed. Still, the PG was in symptom reduction and remission at Y1 and Y5, suggesting that recovery from depression may be associated with improved auditory attention and thus verbal memory performance.

The findings are contrary to previous studies demonstrating an association between symptom severity and cognitive functioning (35). However, they align with Hammar et al. (27) who found no association between symptom severity and verbal memory in recurrent MDD, and with Hammar and Schmid (69) who reported no association between symptom severity and visual memory, but demonstrated memory improvement in remission. Moreover, underlining the importance of symptom load for cognitive functioning, improvement in memory performance is found in remission (13, 14, 69, 95), also for FE MDD (26, 62), and differences in cognitive function is demonstrated between fully remitted, partially remitted, and MDD patients in the acute phase (70). Hence, there is some indication that memory function related to auditory attention show state effects.

### Verbal memory and relapse experience

Exploring the difference in verbal memory function between patients who experienced relapse from Y1 to Y5 and those that did not, revealed no difference between the two groups in performance on the first immediate free recall, trial 1, condition across time. Thus, the results do not show any association between relapse experience and poor auditory attention problems. See **Appendix 1**. for statistical analysis according to relapse experience.

The results are in line with limited studies following FE MDD patients in remission, suggestive of improved verbal memory function in remission (26, 62). Furthermore, comparisons between recurrent and FE MDD have demonstrated verbal memory impairment only in recurrent MDD (56, 61) and that recurrent MDD show larger impairment (45, 55). However, the findings contradict studies reporting more severe cognitive impairment with increasing number of episodes (14, 23, 56). Again, the results highlight a different neurocognitive profile in FE MDD compared to recurrent MDD, suggesting similar patterns of impairment may emerge in FE MDD with sufficient follow-up time and illness duration.

Sample characteristics, primarily age and illness stage, e.g. FE, can contribute to explain absence of scarring effects in the present study. In the current sample, deficits are limited and appear to normalize between episodes. In remission, neurogenesis contributes to cognitive improvement (73, 83) and hinder atrophy and subsequent impairment. However, neurobiological processes underlying depression could impair neurogenesis and cause damage to relevant brain regions, such as the hippocampus, potentially resulting in cognitive impairment. The absence of a lifetime history of recurrent depressive episodes in the present sample may help explain why such neurotoxic effects with subsequent atrophy and functional impairment, are not yet evident (72, 84) but may emerge later in life.

Dysregulated cortisol levels may contribute to increased vulnerability to hippocampal atrophy (82, 83), associated with impaired memory function (84). Antidepressants, commonly used in the present sample, may contribute to normalization of HPA-function and thus impact cortisol levels (76, 81). Increased illness duration leads to prolonged periods of stress and dysregulated cortisol levels (79), found in MDD, including FE MDD (77, 78). However, an association between impaired suppression and cognitive function is observed only in recurrent MDD, not FE MDD (77, 78), suggesting that illness duration and exposure to long-term stress may influence whether cortisol has a damaging impact on the brain. Thus, while such impairment is not evident in the present sample, the possibility of scarring effects emerging over time cannot be ruled out. Early intervention, for instance targeting residual symptoms, is crucial to reduce the risk of recurrent episodes and cognitive deterioration, particularly if scarring effects occur in recurrent MDD, as suggested by previous literature (14, 22, 23, 45, 56).

### Relevance for the trait, state, or scar perspective

The results yield mixed support for the three perspectives. No support was found for the trait perspective, considering deficits normalized in remission; thus, were dependent on illness state (64); besides, confirming the trait perspective requires a different design assessing memory before MDD onset. In contrast, the state perspective was partially supported by the results showing state-specific deficits in the acute phase with normalization in remission, although deficits did not seem to fluctuate with symptom severity (63). Further, the results indicated no scarring effects, as impairment did not worsen with relapse experiences (22). However, ruling out scarring effects requires a longer follow-up. The cognitive deficits found in the present study appears to be state-specific. Nonetheless, state-specific impairment in FE MDD may appear as scar impairment in recurrent MDD due to the neurotoxic duration of depression.

### Strengths and limitations

The present study is the first to longitudinally investigate verbal memory in FE MDD over five-years, contributing to the understanding of the neurocognitive profile in MDD. However, the sample size is relatively small with dropout throughout the study duration. This can potentially make the study underpowered to detect small effect sizes, and multivariate effects, and increase the risk of type 2 errors. Even though the study corrected for multiple comparisons, the possibility of type-1 errors cannot be ruled out, given the number of statistical comparisons in this group. The results concerning deficits in immediate recall trial 1 condition should be considered preliminary until it is replicated. The inability to detect substantial memory impairment may also be explained by test characteristics and admission. The CVLT- II is well-structured, circumstances MDD patients seem to profit from (27). Consequently, non-significant results together with correlations and effect sizes should be interpreted with caution. Yet, a notable strength lies in the repeated symptom assessments, providing a comprehensive understanding of the longitudinal relationship between memory and symptoms. The extended follow-up duration enables insights into changes and stability in cognitive function across various phases of the illness. Additionally, the use of standardized neuropsychological tests facilitates comparisons with other studies.

The sample is well-defined and consists of a selected group which limits the generalizability to other sub-groups, as well as comparability of results with other studies considering different sub-groups of MDD, may show different patterns of memory functioning. Several potential confounding variables may have influenced the results. Further, treatment effects can influence memory function and thus results. Several of the patients received psychological treatment, and approximately half of the patient sample were prescribed antidepressant medication. Although recognized as being small (37), the effects of antidepressants on cognitive function are not fully understood. Moreover, no clinical assessment of the HC was conducted. Hence, potential unknown symptoms in the HC may have affected the results. Age (29, 30), comorbidity (34), and hospital admissions (70) can affect cognitive performance, and so the present study can inform on cognitive functioning in MDD without these variables confounding the result. The sample being FE patients, allows for ruling out potential effects of earlier episodes on cognition, and consequently gain a more concise understanding of the relationship between memory and symptom course. Furthermore, with the inclusion of a matched control group, potential learning effects can be dismissed. The PG and the HC showed comparable IQ scores.

Altogether, the limited sample size, particularly after dividing the PG by experience of relapse, may have contributed to the inability to detect scarring effects. Thus, the results are to be considered preliminary and should be replicated in larger samples. Further, worse cognitive function has been reported in recurrent MDD compared to FE MDD already after two episodes (55), and a longer duration may be necessary as impairment can require time to manifest and worsen with chronicity and severity of affective episodes. Therefore, we cannot dismiss the possibility of persisting impairment in other sub-groups or with a more extended follow-up of the PG. Importantly, the study did not investigate subgroups *within* the current sample according to cognitive deficits. This could have been interesting given a bigger sample, allowing to divide the group according to deficits in verbal memory function, possible targeting patients with special needs. This underscores the need for a longer follow-up of large samples of patients to enhance the understanding of memory deficits and development in a longitudinal perspective.

### Future directions

Future studies exploring memory in MDD should include larger sample sizes and well-defined samples consisting of sub-groups of MDD patients with clear inclusion and exclusion criteria. This may contribute to an improved understanding of potential significant variables such as depression severity, illness phase, depression type, medication, and admission status, apparently lacking in the existing literature. Longitudinal studies with extended follow-ups across different illness phases, ideally starting before the development of full-threshold FE MDD, would provide valuable insights, particularly regarding the trait perspective. Further, incorporating self-report measures of memory is recommended, as subjective reports of cognitive impairment often exceed impairment found in neuropsychological tests (18, 19). Thus, the lack of self-report measures may limit the understanding of memory function in FE MDD.

### Clinical implications

In a clinical perspective, the results may add to the understanding of the neurocognitive profile in FE MDD. Contrary to subjective reports of extensive memory problems in MDD (18, 19), our data showed sparse deficits, indicative of auditory attention deficits in FE MDD patients. However, such auditory attention impairment may impact daily memory function as it typically relies on attention (90) and affecting the learning process which again affect memory. Such processes could align with the commonly reported subjective experience. Thus, despite being relatively small, the identified impairment may influence daily life functioning (17). These results can inform prevention and treatment strategies, addressing impairment while highlighting preserved functions. Additionally, patients can understand and manage their cognitive challenges through repetition and attention-focused strategies, and the knowledge holds significance for the patient’s network, broadening their understanding of cognitive impairment’ impact on daily life. Integrating this understanding into prevention and treatment approaches may help reduce the risk of relapse, emphasizing the importance of early discovery and monitoring of cognitive symptoms, considering some impairments are evident already in FE MDD. Early intervention may prove beneficial for recovery and minimize potential scarring effects which may manifest later in the course of the illness.

## Conclusion

In conclusion, patients with FE MDD show poorer performance in the first trial of immediate free recall in the acute phase of illness, compared to healthy controls, and the performance improved significantly in phases of symptom reduction and remission in a five-year follow-up, reaching the same level as healthy controls; thus, indicating a state-related impairment. The patient group shows intact verbal learning with repetition, which again support their recall -and recognition function. No systematic association between verbal memory function and depression severity was identified across time. Further, no relationship between verbal memory functioning and relapse experience was evident across the five-year follow-up for the patient group, consequently there were no indication of trait or scarring effects in this FE MDD population.

## Data Availability

The raw data supporting the conclusions of this article will be made available by the authors, without undue reservation.
