# Supplementary material for: Verbal memory in major depressive disorder in a long-term perspective: a five-year longitudinal study of first episode patients
Source: Front Psychiatry. 2025 Jul 3;16:1623126. doi: 10.3389/fpsyt.2025.1623126 (PMC12267185; doi:10.3389/fpsyt.2025.1623126)
Supplement: Supplementary file 1 [file Supplementaryfile1.docx]

Appendix 1. for statistical analysis according to relapse experience

Dividing the PG according to relapse experience from inclusion and across time, from T1 to T3, independent samples t-tests revealed no significant differences in performance on the immediate free recall condition at T1, T2 or T3 between patients that experienced relapse (*M* = 7.63, *SD* = 1.67) and those that did not (*M* = 7.50, *SD* = 7.26), *t*(20) = 0.14, *p* = .888 (two-tailed). The mean score difference was 0.13, 95% CI [-1.71, 1.96], η^2^ = .00. Further, there were no significant differences in performance on the immediate free recall condition at T2 between patients that experienced relapse (*M* = 10.56, *SD* = 1.71) and those that did not (*M* = 10.17, *SD* = 2.71), *t*(20) = 0.41, *p* = .685 (two-tailed). The mean score difference was 0.40, 95% CI [-1.61, 2.40], η^2^ = .01. At T3 there were no significant differences in performance on the immediate free recall condition between patients that experienced relapse (*M* = 9.50, *SD* = 2.48) and those that did not (*M* = 9.83, *SD* = 1.94), *t*(20) = -0.30, *p* = .770 (two-tailed). The mean score difference was -0.33, 95% CI [-2.68, 2.02], η^2^ = .00. The independent samples t-test investigating the change score from T1 to T3 showed that it was no significant difference between the patients that experienced relapse (*M* = 1.87, *SD* = 2.47) and those that did not (*M* = 2.33, *SD* = 2.58), *t*(20) = -0.38, *p* = .706 (two-tailed). The mean score difference was -0.46, 95% CI [-2.96, 2.04], η^2^ = .00.
